# Readability of online patient-based information on bariatric surgery

**DOI:** 10.15171/hpp.2019.22

**Published:** 2019-05-25

**Authors:** Zoë Meleo-Erwin, Corey Basch, Joseph Fera, Danna Ethan, Philip Garcia

**Affiliations:** ^1^Department of Public Health, William Paterson University, Wayne, New Jersey 07470, USA; ^2^Department of Mathematics, Lehman College, The City University of New York, Bronx, New York 10468; ^3^Department of Health Sciences, Lehman College, The City University of New York, Bronx, New York 10468, USA

**Keywords:** Obesity, Bariatric surgery, Readability, Health literacy, Online information

## Abstract

**Background:** Web-based patient education literature has been shown to be written at reading levels far above what is recommended. Little is known about the overall readability of current internet-based bariatric surgery information. The purpose of this study was to assess the readability of current bariatric material on the internet.

**Methods: ** The term "weight loss surgery" was searched using the Chrome browser on the first 15pages of URLs that appeared with content written in English. Using five readability measures, scores were generated using Readable.io for written content on a sample of 96 websites. Scores were sorted into the readability categories of "easy," "average," and "difficult."

**Results: ** Almost 93% of websites, both .com and .org, sampled received an unacceptable readability score on each assessment.

**Conclusion:** Accurate and appropriate information about bariatric procedures is critical for patient comprehension and adherence to recommended protocols.

## Introduction

The medical community considers weight loss surgery (also known as bariatric surgery) to be both the most efficacious and the most durable intervention for both weight loss and resolution of metabolic diseases.^1^ Between 1998 and 2004, the number of bariatric procedures performed in the United States increased by 726% for individuals between the ages of 18–54, and nearly 2000% for those between the ages of 55-64.^2^ Approximately 200 000-250 000 Americans undergo bariatric surgery each year,^3,4^ although some research suggests that these numbers have plateaued.^5,6^

Given the overall rapid increase in bariatric procedures, weight loss surgery has generated substantial public interest and attention. The internet is a primary site of information for individuals who desire weight loss in general,^7^ and by means of a bariatric procedure in specific.^8,9^ Individuals medically classified as overweight or obese may be particularly inclined to search the internet to learn more about health and weight given of the pervasive stigma associated with larger body sizes.^10^ The internet may thus serve as a key site of information for those who prefer to seek information on weight and health anonymously.^11^

However, individuals do not simply passively take in e-information on weight loss methods but actively make decisions based upon what they read online. Estimates suggest that nearly half ^11^ to more than three quarters ^9^ of individuals interested in weight loss surgery conduct online searches to gain information. Roughly 25% of these individuals decide to pursue a bariatric procedure based on information found online.^9^ Moreover, following a bariatric procedure, high numbers of patients continue to use the internet for post-operative information and support.^9^

Bariatric surgeries are not procedures to be undertaken lightly. Though the in-hospital mortality rate is low at approximately 0.10%,^5,12,13^ complications occur with more frequency, with some estimates putting the rate between 7.6%^6^ and 10%.^14^ Moreover, in order to reduce the risk of complications and avoid or reduce side effects, patients must strictly follow life-long post-operative eating and nutritional supplementation directives from their home bariatric clinics. Finally, weight regain following a bariatric procedure, though far less common than with dieting, is nevertheless common within the bariatric population.^15-19^ With that said, the amount of regain does vary by bariatric procedure type.^18^ To address patient regain, conversion from one bariatric procedure to another has increased.^19^ Such re-operative procedures are associated with higher rates of adverse events than are the primary bariatric surgeries.^19^ Patient understanding of the risks of bariatric surgeries and the requirements of living with them is thus imperative in order to minimize the risk of side effects, regain, and complications.

However, a number of studies ^11,20-23^ have found that the quality of weight loss and bariatric surgery advice shared online is variable, with much of it being inaccurate and lacking professional input. Notably, two studies^11,24^ have found that the quality of online information about the risks associated with bariatric procedures is quite poor. Other research^25^ has determined that conflict of interest is present in over 75% of bariatric websites. Given that bariatric patients forget or misremember key aspects of clinical preoperative patient education one year following bariatric surgery, post-operative patients may be particularly vulnerable to poor quality information shared online.^26^

In addition to the accuracy of the information on bariatric surgery online, there is some evidence to suggest that web-based patient education materials are written at a reading level far above recommendations at or below sixthgrade.^27^ For instance, materials available on the American Society for Metabolic and Bariatric Surgery (ASMBS) website meant for patients were determined to be written at a 15th grade level.^28^ A similar study scored the ASMBS site at the 17th grade level.^29^ A 2004 study of 40 bariatric websites found that the average grade level of the sites was 11.1.^25^ Given that the only study,^25^ to the best of the authors’ knowledge, published on the readability of online bariatric patient information was published 15 years ago, the purpose of this study was to provide a more current assessment of internet-based material on bariatric surgery.

## Materials and Methods

The methods for this study were based on prior research on readability.^30,31^ Using Chrome as a browser, the term “weight loss surgery” was searched on the first 15 pages of URLs that appeared with content written in English. It should be noted that prior to searching, the browser cache, cookies, and history were cleared. A total of 49 websites were excluded in the search process due to splash pages/external links only, location advertisements, and no pertinent information related to weight loss surgery. Hence, a sample size of 96 websites was reached.

Readability scores were generated for written content on the website using Readable.io, a National Institutes of Health recommended program.^32^ The following measures of readability were used: Flesch-Kincaid Grade Level (FKGL), Gunning Fog Index (GFI), Coleman-Liau Index (CLI), Simple Measure of Gobbledygook (SMOG) Grade Level, and Flesch-Kincaid Reading Ease (FRE). The scores for grade level reading tests were sorted into readability categories. Easy was considered to be lower than grade 6, average was considered to be between grades 6 and 10, and difficult was considered to be greater than grade 10. The FRE was grouped as easy being a score of 80 to 100, average being a score of 60 to 79, and difficult being a score of 0 to 59.

The extensions of all URLs were recorded for comparisons to determine where information was derived.

### Statistical analysis

Microsoft Excel was used to analyze the data in this study. The analysis was performed on a sample (n=96) of weight loss surgery websites from which five different readability scores were calculated using five different readability tests (FRE, FKGL, GFS, CLI, and SMOG). It is recommended that health materials be written at the fifth or sixth grade level.^27^ Thus, the analysis assumes that an acceptable readability level is indicated by an FRE score greater than or equal to 80.0 or less than or equal to 6.9 on any of the other assessments. To test the claim that weight loss surgery websites are written at an acceptable level, one-sample independent t-tests (α = 0.05, *df* = 95) were performed for each readability assessment. In addition, in order to determine whether or not information written on websites with .com and .org extensions are at differing readability levels, two-sample independent *t* tests (α = 0.05) were calculated.

Studies not involving human subjects are expect from review by the IRB at William Paterson University.

## Results

The analysis overwhelmingly indicates that website material on weight loss surgery is not presented at an acceptable reading level. Out of the 96 sites analyzed, none received an acceptable score on all five assessments used. This, along with the distribution of readability scores both by test and difficulty level, is shown in Table 1. In addition, nearly 93% of the websites sampled qualified as unacceptable in terms of readability score on each of the five assessments.

The average scores for each readability test along with associated standard deviations are shown in Table 2. As indicated in Table 2, the given *P* values are substantially below 0.05. Hence, with strong statistical support, it is highly unlikely that weight loss surgery websites are being written at the level recommended.

The distribution of type of websites sampled largely belonged to two categories. In total, 45 of the 96 websites sampled were .org extensions while 43 out of the 96 sites were .com extensions. Table 3 shows the average readability scores for each of the five assessments for the .com and .org websites sampled separately. When analyzed separately, the .com websites and the .org websites both had unacceptable average readability scores on each of the five assessments.

No significant statistical difference was found between the .com and .org websites. Thus, the information contained on both types of websites was equally likely written at the same unacceptable level. The *P* values are also given in Table 3. A marginal statistical difference was uncovered between the two website types for the CLI and FRE assessments.

The average readability score by search result page was calculated (data not shown). There does not appear to be any strong correlation between search result page and readability. Note that the number of samples per page differed due to exclusion criteria.

## Discussion

The findings of this study indicate that materials on weight loss surgery are written at grade levels that may make it difficult for the general public to understand. This was true both for websites that may be more commercial in nature (.com) and those associated non-commercial entities, including not-for-profit organizations (.org). Although the nature of the internet is that the information contained therein is ever-changing, this study confirms the results of a previous 2004 study^25^ on this topic which found that the average grade level of weight loss surgery websites was 11.1.

This continuity of findings does more than lend credence to the accuracy of Nichols and Oermann’s conclusions^25^ and our own. Rather it suggests that the online information on bariatric surgery is no more accessible to those with lower levels of health literacy today than it was nearly a decade and a half ago. Given the need to adhere to clinical protocols for diet, nutritional supplementation, and exercise is lifelong for this patient population, the authors find this lack of improvement in readability to be highly troubling. As noted, substantial numbers of individuals seeking weight loss by means of bariatric surgery access the internet for information and support both pre- and post-operatively.^9,11^ It is thus imperative that, at a minimum, information on weight loss surgery found on the websites of bariatric clinics and the portion of the ASMBS site geared toward lay individuals be written at the recommended reading level of at or below sixthgrade.^27^

There are number of limitations with this study. First, this study is limited by the cross-sectional design chosen by the authors. Second, because materials were restricted to English, this study cannot comment on the readability of online bariatric information written in other languages. Third, the authors chose a cut-off of 15 pages for the search, however it is possible that searches that continued past this arbitrary cut off would have produced different results. Finally, as noted above, online information changes frequently and it is possible that future studies will find different results. It is our hope that this will be the case. Nevertheless, this study provides an updated assessment of the readability of online bariatric literature.

Bates et al argue that the “digital divide” is more than differential access to technology.^33^ Rather, it is also a divide in the ability to understand information found online. Materials on weight loss surgery must be written at grade levels that may make easy for the general public to understand. It is critical that bariatric candidates comprehend risks associated with weight loss surgery and the psychosocial and behavioral requirements of living with such procedures. Without this, such individuals cannot provide fully informed consent and may be at increased risk for postoperative side effects, complications, and regain.

## Ethical approval

The IRB at William Paterson University does not review studies that do not involve human subjects considering them to be exempt.

## Competing interests

The authors declare that they have no competing interests.

## Authors’ contributions

ZME researched the literature, wrote the literature review, co-wrote the conclusion, and co-conceived the study. CB co-conceived the study, designed the methods, and co-wrote the conclusion. PG had primary responsibility for data collection and assisted with editing the final manuscript. JF analyzed the data. CB, JF, and DE wrote the methods and results sections. All authors reviewed and edited the manuscript and approved the final version.

**Table 1:** Distribution of readability scores by assessment and level

**Readability scores**	**No. of websites (n=96)**
FRE	
Easy (80-100)	0
Average (60-79)	13
Difficult (0-59)	83
FKGL	
Up to grade 6	1
Grades 6-10	53
Beyond grade 10	42
GFI	
Up to grade 6	3
Grades 6-10	25
Beyond grade 10	68
CLI	
Up to grade 6	0
Grades 6-10	12
Beyond grade 10	84
SMOG	
Up to grade 6	0
Grades 6-10	7
Beyond grade 10	89

FRE, Flesch-Kincaid Reading Ease; FKGL, Flesch-Kincaid Grade Level; GFI, Gunning Fog Index; SMOG, Simple Measure of Gobbledygook; CLI, Coleman-Liau Index.

**Table 2:** Mean and standard deviation measures

**Readability test**	**Mean**	**Standard deviation**	***P *** **value**
FKGL	9.78	1.85	1.36E-27
GFI	11.27	2.55	1.75E-30
CLI	12.13	1.86	2.20E-47
SMOG	12.43	1.61	5.35E-55
FRE	47.49	10.76	4.70E-50

FRE, Flesch-Kincaid Reading Ease; FKGL, Flesch-Kincaid Grade Level; GFI, Gunning Fog Index; SMOG, Simple Measure of Gobbledygook; CLI, Coleman-Liau Index.

**Table 3:** Readability test results by website type.

**Readability test**	**.com**	**.org**	***P *** **value**
FKGL	9.64	9.93	0.4721
GFI	11.53	11.27	0.6276
CLI	11.73	12.49	0.0630
SMOG	12.45	12.46	0.9649
FRE	50.05	45.54	0.0500

FRE, Flesch-Kincaid Reading Ease; FKGL, Flesch-Kincaid Grade Level; GFI, Gunning Fog Index; SMOG, Simple Measure of Gobbledygook; CLI, Coleman-Liau Index.
